# Serum exosomal and serum glypican-1 are associated with early recurrence of pancreatic ductal adenocarcinoma

**DOI:** 10.3389/fonc.2022.992929

**Published:** 2022-10-14

**Authors:** Juan Zhao, Madi Guo, Yushuai Song, Shan Liu, Ran Liao, Yu Zhang, Yumin Zhang, Qi Yang, Yuanlong Gu, Xiaoyi Huang

**Affiliations:** ^1^ Biotherapy Center, Harbin Medical University Cancer Hospital, Harbin, China; ^2^ Department of interventional oncology, Taizhou Municipal Hospital, Taizhou, Zhejiang, China; ^3^ NHC Key Laboratory of Cell Transplantation, Harbin Medical University, Harbin, Heilongjiang, China

**Keywords:** pancreatic ductal adenocarcinoma, exosome, glypican 1, diagnosis, early recurrence

## Abstract

**Background:**

The diagnostic performance and prognostic value of serum exosomal glypican 1 (GPC-1) in pancreatic ductal adenocarcinoma (PDAC) remain controversial. In this study, we detected serum exosomal GPC-1 using enzyme-linked immunosorbent assay (ELISA) and determined whether it serves as a predictor of diagnosis and recurrence for early-stage PDAC.

**Methods:**

Serum samples were obtained from patients with 50 PDAC, 6 benign pancreatic tumor (BPT), or 9 chronic pancreatitis (CP) and 50 healthy controls (HCs). Serum exosomes were isolated using an exosome isolation kit. Exosomal and serum GPC-1 levels were measured using ELISA. The freeze–thaw process was carried out to analyze the stability of GPC-1. Receiver operating characteristic (ROC) analysis was employed to assess the diagnostic value of GPC-1. Kaplan–Meier and multivariate Cox analyses were used to evaluate the prognostic value of GPC-1.

**Results:**

The average concentrations of serum exosomal and serum GPC-1 were 1.5 and 0.8 ng/ml, respectively. GPC-1 expression levels were stable under repeated freezing and thawing (d1-5 freeze–thaw cycles vs. d0 P > 0.05). Serum exosomal and serum GPC-1 were significantly elevated in patients with PDAC compared with HCs (P < 0.0001) but were slightly higher compared with that in patients with CP and BPT (P > 0.05). The expression levels of exosomal and serum GPC-1 were elevated 5 days after surgery in patients with PDAC, CP, and BPT (P < 0.05). Patients with high levels of exosomal and serum GPC-1 had a shorter relapse-free survival (RFS) (P = 0.006, and P = 0.010). Multivariate analyses showed that serum exosomal and serum GPC-1 were independent prognostic indicators for early RFS (P = 0.008, and P = 0.041).

**Conclusion:**

ELISA is an effective and sensitive method to detect exosomal and serum GPC-1. The detection of GPC-1 was stable under repeated freezing and thawing cycles and could distinguish early-stage PDAC from HCs but not CP and BPT. Exosomal and serum GPC-1 may be good independent predictors of early recurrence in early-stage PDAC.

## Introduction

Pancreatic cancer (PC) is one of the most lethal cancers worldwide, with 5-year overall survival (OS) rates of less than 6% (1). Approximately 90% of PC cases are pancreatic ductal adenocarcinoma (PDAC) (2). Currently, the diagnosis of PDAC is mainly based on clinical symptoms (3), imaging features (4), and serum markers such as CA19-9 (5). However, more than 50% of patients are diagnosed with PDAC in the advanced stage and miss the opportunity for surgery (6). Even in patients with resectable PDAC, 76.7% of cases recur after a short recurrence-free interval, which is less than 12 months postsurgery (7). Therefore, finding new biomarkers to diagnose PDAC at an early stage or to predict the early recurrence of PDAC is urgently needed.

Glypican-1 (GPC-1) is a cell surface proteoglycan that is upregulated in some types of human cancers, such as breast cancer (BC) (8), esophageal squamous cell cancer (ESCC) (9), and PC (10). Some studies reported that high GPC-1 expression was associated with poor outcomes of PC (10) and glioma (11), and the loss of GPC-1 results in reduced tumor growth, angiogenesis, and metastasis (12). Moreover, Melo et al. reported that GPC-1+ circulating exosomes could distinguish early PDAC patients from healthy controls (HCs) and benign pancreatic disease (BPD) patients with a nearly perfect area under the curve (AUC) of 1.0 (13). After that, the diagnostic efficacy of GPC-1+ exosomes in PDAC was also demonstrated in some other studies, which suggested that GPC-1+ exosomes could not discriminate PDAC from BPD. The diagnostic value of GPC-1 in PDAC remains controversial. In these studies, the most common method used to detect GPC-1+ exosomes was flow cytometry or some new technologies, such as nanosized molecular beacons with high luminescence efficiency and glypican-1-antibody-conjugated Gd-Au nanoclusters (14–17), which require expensive equipment and skilled labor and are not suitable for wide application in the clinic. Enzyme-linked immunosorbent assay (ELISA) is one of the most commonly used serological methods in the clinic owing to its high efficiency, wide availability, and low cost. Developing an ELISA-based method is especially ideal for GPC-1+ evaluation while fully utilizing handy equipment.

In this study, we quantified serum exosomal and serum GPC-1 expression levels using ELISA. Moreover, we evaluated GPC-1 stability under repeated freezing and thawing cycles. We further explored whether serum exosomal and serum GPC-1 could be used as diagnostic markers and prognostic predictors for early-stage PDAC.

## Materials and methods

### Patients and serum samples

From May 2020 to May 2021, 50 patients pathologically diagnosed with stage I-II PDAC, 9 patients with CP, 6 patients with BPT (4 pancreatic serous cystadenomas and 2 pancreatic benign cysts), and 50 HCs matched for age and sex were enrolled at Harbin Medical University Cancer Hospital. The inclusion criteria were as follows: (a) ≥18 years; (b) diagnosed with pancreatic ductal carcinoma, cystic lesions, and serous cystadenomas by histopathological examination; (c) diagnosed with CP *via* clinical, imaging and pathological examination(destruction of normal pancreatic architecture, acinar atrophy, and fibrosis). (d) no anticancer treatment, such as chemotherapy or radiotherapy, before surgery; (e) absence of other malignancies; and (f) available clinical records. The TNM stage was assessed based on the AJCC 8th Edition for pancreatic cancer. All patients were followed up through April 30, 2022. Recurrence-free survival (RFS) was defined as time from the date of surgery until the first confirmed recurrence. Ethics approval was provided by the Ethics Committee of Harbin Medical University Cancer Hospital (2019-118-IIT), and written informed consent for use of the clinical samples in this study was obtained from each patient.

Five-milliliter venous blood samples were acquired from both patients and HCs in the fasting state. Patient serum samples were collected before surgery and again 5 days after surgery. Serum samples (1.5-2 ml) were collected and then centrifuged at 3000 × g for 15 minutes at 4°C to remove cellular debris (within 2 hours of collection). Serum supernatants were collected and stored frozen at -80°C until use.

### Freeze–thaw cycles

To assess the impact of repeated freeze–thaw cycles (f/t) on serum exosomal and serum GPC-1, 3 serum samples from patients with pancreatic diseases were used. To avoid multiple sample collection, each serum sample was divided into six aliquots and stored at -80°C. One aliquot was immediately subjected to exosome extraction and ELISA determination, assigned as the d0 f/t cycle sample. The other aliquots were subjected to the following treatments: (a) one aliquot remained frozen at -80°C until analysis, assigned as the d1 f/t cycle sample; (b) four aliquots exposed to repeated freeze–thaw cycles for two, three, four, and five cycles were assigned as the d2-5 f/t cycle samples. Any one f/t cycle sample was kept at 4°C until completely melted and then frozen at -80°C again. After all f/t cycles were completed, exosomes were extracted from all of the serum samples, and GPC-1 concentrations were measured.

### Isolation and characterization of serum exosomes

Exosomes were extracted using ExoQuick^®^ (EQULTRA-20A-1, SBI) according to the operation manual. Briefly, serum samples were thawed and then centrifuged at 3000 × g for 15 minutes at 4°C to remove cellular debris. An aliquot of 250 µL supernatant was placed into a 1.5 mL enzyme-free centrifuge tube containing 67 µL of ExoQuick. The mixture was vortexed and incubated at 4°C for 30 minutes and then centrifuged at 1500 × g for 30 minutes at 4°C to precipitate the beige exosome pellet at the bottom of the tube. After removal of the supernatant, the obtained exosomes were suspended in 50 µL of PBS.

The serum exosomes were morphologically analyzed by transmission electron microscopy (TEM). Briefly, exosome suspension was dropped onto formvar-carbon-coated grids and stained with 3% phosphotungstic acid solution. TEM images were captured using a Hitachi HT7700 transmission electron microscope (Japan). The size distribution of exosomes was determined by Flow Nano Analyzer model type N30 (NanoFCM Inc., Xiamen, China). For Western blotting analysis, serum exosomes were treated with RIPA lysis buffer (Beyotime, China) with a protease inhibitor (Roche, USA), and the protein concentration was determined with BCA reagent (Beyotime, China). Protein samples (20 µg) were separated by 12.5% SDS–PAGE (Epizyme, China) and then transferred onto PVDF membranes (Roche, Germany). The membranes were probed with primary antibodies against CD9, CD81, and TSG101 (1:1000, ab275018, Abcam, UK) at 4°C overnight and then incubated with secondary antibodies (Absin, China) for 1 hour. The protein bands were visualized using a chemiluminescence imaging system (ProteinSimple, USA).

### ELISA

Serum samples were thawed and centrifuged again at 3000 × g for 15 minutes at 4°C to remove cells and debris. Human GPC-1 ELISA kits were obtained from Jianglai Bioscience (JL19652). Experiments were performed according to the manufacturer’s instructions. Briefly, 50 µL of serum, serum exosomal suspension, and standard solutions were added to a GPC-1 antibody-coated 96-well plate. Subsequently, HRP-labeled capture antibodies were added and then incubated for 1 hour at 37°C. Next, the plates were washed 5 times, and TMB substrate solution was added. The reaction was terminated by the addition of a sulfuric acid solution, and the color intensity was measured spectrophotometrically at a wavelength of 450 nm (Multiskan™ FC, Thermo Fisher, USA). The GPC-1 concentration in the samples was determined by comparing the optical density of each sample to a standard curve. Each plate test was repeated three times.

### Bioinformatics analysis based on public databases

The transcription and protein expression levels of GPC-1 were acquired from normal tissues and cancer tissues from the GEPIA (http://gepia.cancer-pku.cn/ ) (18) and UALCAN databases (ualcan.path.uab.edu/home) (19). Kaplan–Meier plotter (http://kmplot.com/analysis/) (20) was used to obtain the hazard ratio (HR) with its corresponding 95% confidence interval (CI) from the Cox proportional hazards model to analyze the prognostic value of GPC-1 expression for RFS in pancreatic cancer.

### Statistical analysis

Differences between GPC-1 concentrations were evaluated by Student’s t test, with P < 0.05 considered significant. The receiver operating characteristic (ROC) curve was used to evaluate the diagnostic power of GPC-1 for early-stage pancreatic cancer and to determine the optimal cutoff values of GPC-1 based on the maximum Youden index. The chi-square (χ2) test was performed to analyze the associations between GPC-1 expression and clinicopathological features. Survival was evaluated using the Kaplan–Meier survival method and compared between groups using log‐rank statistics. The diagnostic and survival predictions were evaluated by the area under the receiver operating characteristic curve (AUC). Univariate and multivariate analyses were performed using Cox regression analysis, and P < 0.05 was considered statistically significant.

## Results

### Serum exosomal and serum GPC-1 were measured by ELISA

**Figure 1:**
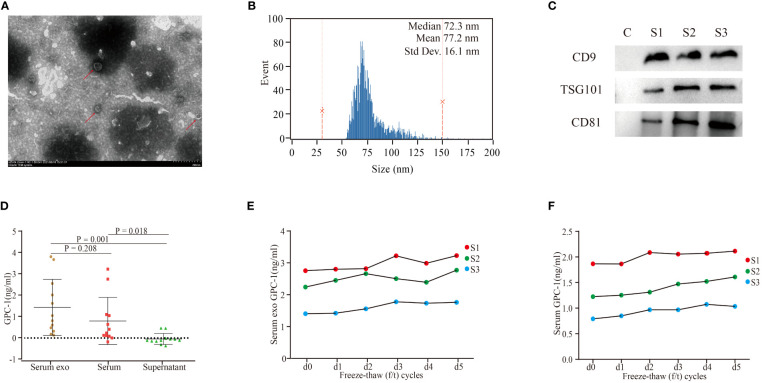
Characterization of serum exosomes and GPC-1 expression in serum exosomes and serum. **(A)** TEM image of exosomes extracted from the serum of patients with PDAC. **(B)** Nano flow cytometry analysis of serum exosomes. **(C)** Western blot analysis of the serum exosomal markers CD9, TSG101, and CD81. TEM, transmission electron microscopy; PDAC, pancreatic ductal carcinoma. **(D)** Serum exosomal, serum and exosome-depleted serum supernatant GPC-1 expression levels. Serum exosomal **(E)** and serum **(F)** GPC-1 expression levels were stable under 1-5 freeze–thaw cycles.

Exosomes were successfully extracted from the serum of patients. TEM clearly showed sizes with a diameter of ~100 nm and morphology with a classical complete membrane structure of an extracellular vesicle (**Figure 1A**). Nano flow analysis showed that the vesicles isolated from serum were mainly approximately 72.3 nm in diameter (77.2 ± 16.1 nm, **Figure 1B**). Western blot assays showed that TSG101, CD81, and CD9 were present in the samples randomly selected from each group (**Figure 1C** **).** These results confirmed that the collected extracellular vesicles were exosomes.

As shown in **Figure 1D**, serum exosomal and serum GPC-1 concentrations were 1.5 ± 1.3 ng/ml and 0.8 ± 1.1 ng/ml, respectively. Serum exosomal GPC-1 was slightly higher than serum GPC-1, but the difference was not statistically significant (P = 0.208). The expression of GPC-1 was enriched in the exosome fraction and almost absent in the exosome-depleted supernatant fraction (exosomal GPC-1 vs. serum exosome-depleted supernatant GPC-1 P = 0.001). Serum exosomal and serum GPC-1 expression levels were stable under 1-5 f/t cycles (**Figures 1E, F** **;** GPC-1 d1-5 f/t cycles vs. GPC-1 d0 f/t cycle P > 0.05). These results confirmed that serum exosomes were detectable by ELISA and stable under repeated freeze–thaw (f/t) processes.

### Expression levels of serum exosomal and serum GPC-1 in patients with pancreatic cancers and HCs

There were 50 PDAC, 9 CP, and 6 BPT patients and 50 HCs enrolled in the study. The age, sex, CA19-9, differentiation, tumor size, lymph node metastasis, and TNM stage of the patients are presented in **Table 1**. Patients with PDAC had an average age of 60.9 ± 8.8 years, with 56.0% of patients being male, and those with nonpancreatic cancer had an average age of 55.2 ± 9.3 years, with 53.3% of patients being male. According to our chi-square (χ2) test, there was no significant difference in baseline characteristics such as age, sex, location and tumor size between the comparable groups.

**Table:** Table 1 Characteristics of patients and healthy controls enrolled in the study.

Characteristic	PDAC	CP and BPT	HCs	P value (PDAC vs. CP and BPT)	P value (PDAC vs. HCs)	P value (CP and BPT vs. HCs)
Sex	Cases (%)	Cases (%)	Cases (%)	0.855	0.841	0.964
Male	28 (56.0)	8 (53.3)	27 (54.0)			
Female	22 (44.0)	7 (46.7)	23 (46.0)			
Age (years)				0.415	0.841	0.341
≤ 60	24 (48.0)	9 (60.0)	23 (46.0)			
> 60	26 (52.0)	6 (40.0)	27 (54.0)			
CA19-9				0.0002	<0.0001	0.155
≥37	37 (74.0)	3 (20.0)	4 (8.0)			
< 37	13 (26.0)	12 (80.0)	46 (92.0)			
Location:				0.177		
Head	33 (66.0)	7 (46.7)				
Body or Tail	17 (34.0)	8 (53.3)				
Tumor size				0.225		
> 4cm	12 (24.0)	6 (40.0)				
≤ 4cm	38 (76.0)	9 (6.00)				
Differentiation						
Well and Moderate	25 (50.0)					
Poor	25 (50.0)					
Nerve invasion						
Yes	35 (70.0)					
No	15 (30.0)					
Lymph nodes						
Yes	12 (24.0)					
No	38 (76.0)					
TNM stage						
I	27 (54.0)					
II	23 (46.0)					

PDAC, pancreatic ductal adenocarcinoma; CP, chronic pancreatitis; BPT, benign pancreas tumors; HCs, heathy controls.

**Figure 2:**
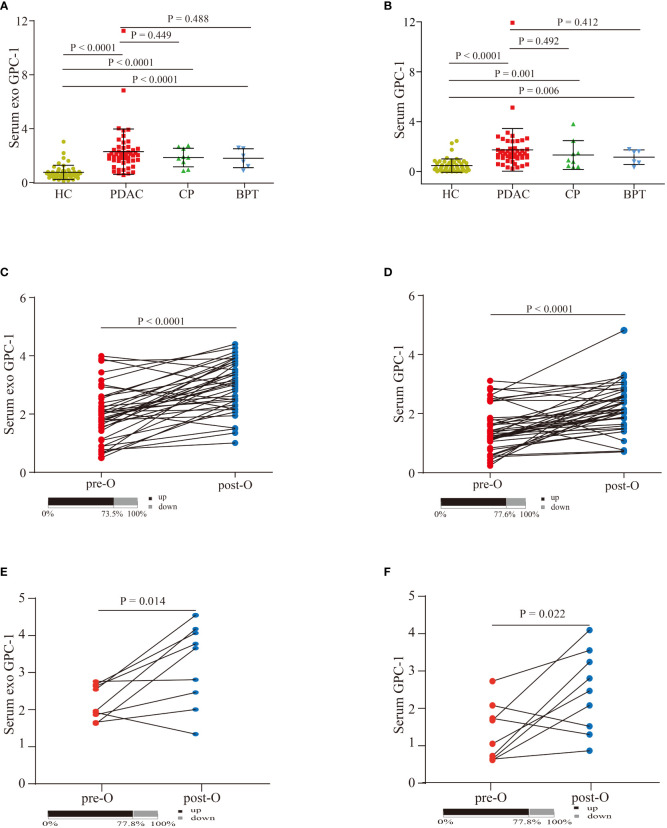
Serum exosomal and serum GPC-1 expression levels in patients with pancreatic diseases and HCs. **(A)** Serum exosomal GPC-1 levels in HCs and PDAC, CP, and BPT patients. **(B)** Serum GPC-1 expression levels in HC and PDAC, CP, and BPT patients. Preoperative and postoperative serum exosomal GPC-1 expression levels **(C)** and serum GPC-1 expression levels **(D)** in patients with PDAC. Preoperative and postoperative serum exosomal GPC-1 expression levels **(E)** and serum GPC-1 expression levels **(F)** in patients with CP and BPT. PDAC, pancreatic ductal carcinoma; HCs, healthy controls; CP, chronic pancreatitis; BPT, benign pancreatic tumor.

As shown in **Figures 2A, B**, the serum exosomal GPC-1 expression levels were significantly increased in patients with PDAC compared with HCs (2.3 ± 1.7 ng/ml vs. 0.7 ± 0.5 ng/ml; P < 0.0001). The average levels of serum GPC-1 were also significantly elevated in PDAC patients compared with HCs (1.7 ± 1.7 ng/ml vs. 0.5 ± 0.5 ng/ml; P < 0.0001). In addition, GPC-1 expression levels were increased in patients with CP and BPT compared with HCs (both P < 0.0001). Serum exosomal and serum GPC-1 expression were slightly lower in patients with CP and BPT than in those with PDAC, but the differences were not statistically significant (P = 0.449, P = 0.488; P = 0.492, and P = 0.412, respectively). Compared with the preoperative serum exosomal and serum GPC-1 levels, the expression levels of postoperative serum exosomal and serum GPC-1 were significantly higher in patients with PDAC (**Figures 2C, D**; 73.5% higher in serum exosomal GPC-1 with P < 0.0001 and 77.6% higher in serum GPC-1 with P < 0.0001). Interestingly, patients with CP and BPT also had elevated postoperative serum exosomal and serum GPC-1 levels (**Figures 2E, F** **;** 77.8% higher in serum exosomal and serum GPC-1 with P = 0.014 and P= 0.022). These results indicated that serum exosomal and serum GPC-1 increased in patients with pancreatic diseases, including PADC, CP and BPT, and pancreatic surgery also led to an upregulation of GPC-1 expression.

### Diagnostic value of serum exosomal and serum GPC-1 for early-stage PDAC

**Figure 3:**
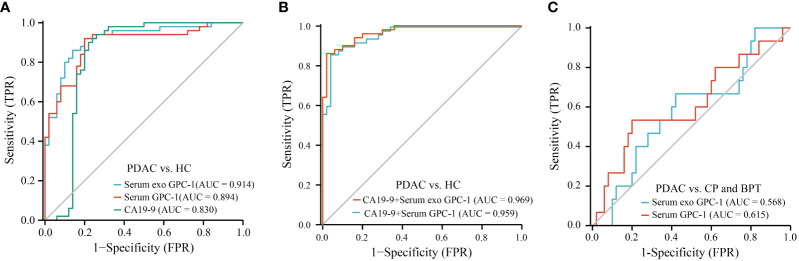
ROC curves for GPC-1 and CA19-9 in the diagnosis of early-stage PDAC. **(A)** ROC curve analyses of serum exosomal GPC-1, serum GPC-1, and CA19-9 as parameters to discriminate PDAC patients from HCs. **(B)** Combinations of serum exosomal GPC-1 and CA19-9 and serum GPC-1 and CA19-9 resulted in higher AUC values to distinguish patients with PDAC from HCs. **(C)** ROC curve for serum exosomal GPC-1and serum GPC-1 in patients with PDAC vs. CP and BPT. ROC, receiver operating characteristic; AUC, area under the receiver operating characteristic curve; PDAC, pancreatic ductal carcinoma; HCs, healthy controls; CP, chronic pancreatitis; BPT, benign pancreatic tumor.

ROC curves were used to assess the diagnostic value of preoperative GPC-1 and CA19-9 for PDAC. The AUC was 0.914 for serum exosomal GPC-1 (**Figure 3A**, P < 0.0001, cutoff > 1.405 ng/ml, sensitivity: 92.0%, specificity: 80.0%) and 0.894 for serum GPC-1 (**Figure 3A** **;** P < 0.0001, cutoff > 1.035 ng/ml, sensitivity: 92.0%, specificity: 80.0%). In contrast, the AUC of CA19-9 was 0.830 (**Figure 3A**; P < 0.0001, cutoff > 33.84 U/ml, sensitivity: 92.0%, specificity: 76.0%). We further evaluated the diagnostic value of GPC-1 in combination with CA19-9. The combination of serum exosomal GPC-1 and CA19-9 improved the diagnostic accuracy for early-stage PDAC (**Figure 3B**; AUC 0.969, 95% CI 0.941-0.996, P < 0.001, cutoff > 1.194, sensitivity: 86.0%, specificity: 98.0%). The combination of serum GPC-1 and CA19-9 could discriminate early-stage PDAC patients from HCs with an AUC of 0.959, 86.0% sensitivity and 96.0% specificity (**Figure 3B**; P < 0.001, 95% CI 0.926-0.992, cutoff > 1.131). However, neither serum exosomal nor serum GPC-1 could distinguish PDAC from CP and BPT (**Figure 3C**; AUCs of 0.568, P = 0.427, 95% CI 0.406-0.730, and 0.615, P = 0.181, 95% CI 0.442-0.788, respectively). These findings indicated that serum exosomal and serum GPC-1 could distinguish PDAC from HCs but not from CP and BPT.

### Prognostic value of serum exosomal and serum GPC-1 for patients with early-stage PDAC

**Figure 4:**
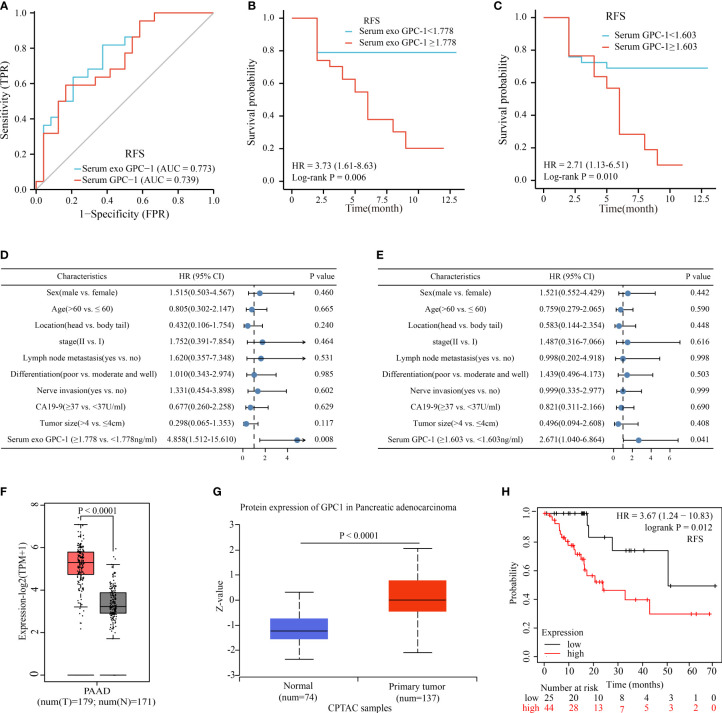
GPC-1 was correlated with RFS in patients with PDAC. **(A)** ROC curve analyses of serum exosomal GPC-1 and serum GPC-1for RFS in early-stage PDAC. Kaplan–Meier curves for RFS according to the optimal cutoff value of serum exosomal GPC-1 **(B)** and serum exosome GPC-1 **(C)** in early-stage PDAC patients. Multivariate survival analysis of serum exosomal GPC-1 **(D)** and serum GPC-1 **(E)** expression for RFS in patients with PDAC. The mRNA **(F)** and protein **(G)** expression levels of GPC-1 were significantly increased in cancer tissues. **(H)** Kaplan–Meier curves for RFS according to the expression of GPC-1 in PDAC tissues. PAAD, pancreatic cancer; PDAC, pancreatic ductal carcinoma; ROC, receiver operating characteristic; RFS, relapse-free survival. .

As shown in **Figure 4A**, the optimal cutoff values for preoperative serum exosomal GPC-1 and serum GPC-1 were 1.778 ng/ml (AUC = 0.773, P < 0.001, 95% CI: 0.637-0.908, sensitivity = 81.8%, specificity = 62.5%) and 1.603 ng/ml (AUC = 0.739, P < 0.001, 95% CI: 0.594-0.883, sensitivity = 59.1%, specificity = 83.3%), respectively. According to the optimal cutoff values, the patients were divided into low (< 1.778 ng/ml) and high (≥ 1.778 ng/ml) serum exosomal GPC-1 groups and low (< 1.603 ng/ml) and high (≥ 1.603 ng/ml) serum GPC-1 groups. Then, we evaluated the associations between these indexes and clinicopathological features. Preoperative serum exosomal and serum GPC-1 were not related to age, sex, CA19-9, location, tumor size, differentiation, nerve invasion, lymph node metastasis, or TNM stage (**Supplementary Table 1**). Next, Kaplan–Meier analysis was conducted to identify the prognostic significance of GPC-1, in which shorter RFS was demonstrated to be significantly associated with high levels of preoperative serum exosomal GPC-1 (**Figure 4B**; P = 0.006) and serum GPC-1 (**Figure 4C**; P = 0.010). Furthermore, RFS related factors were further analyzed using univariate and multivariate analysis. By univariate analysis, we found only serum exosomal and serum GPC-1, but not the clinical and pathological features, were significantly associated with early RFS (**Supplementary Table 2**, P = 0.017 and P = 0.021). Likewise, in the multivariable model, the returned readouts showed only the preoperative serum exosomal and serum GPC-1 were significant prognostic factors for RFS in PDAC patients (**Figures 4D, E**; P = 0.008 and P = 0.041).

The GEPIA and UALCAN databases were used to evaluate the expression level of GPC-1 in PAAD. Compared to normal pancreatic tissues, the mRNA and protein expression levels of GPC-1 were significantly upregulated in cancer tissues (**Figures 4F, G**; P < 0.0001 and P < 0.0001, respectively). Then, the relationship between GPC-1 and prognosis was established using Kaplan–Meier Plotter. The results indicated that high expression of GPC-1 was correlated with poor RFS in PDAC (**Figure 4H** **;** P =0.012). These results collectively suggested that GPC-1 could be used as an independent predictor for recurrence in patients with early-stage PDAC.

## Discussion

GPC-1, a member of the heparan sulfate proteoglycan (HSPG) family, can act as a coreceptor for growth factors and plays a role in cellular signaling, mainly including fibroblast growth factors (FGFs), vascular endothelial growth factor-A (VEGF-A), transforming growth factor-β (TGF-β), Wnt, bone morphogenic protein (BMP), and Hedgehog (Hh) (12, 21). For example, FGF2 can trigger the formation of new blood vessels to provide nutrients and oxygen for cancers and block programmed cell death through both autocrine and paracrine signaling (22), which may be a key factor that promotes tumors in the tumor microenvironment. Sparn’s study found that GPC-1 could drive the unconventional secretion of FGF2 (23). Huang’s study showed that GPC-1 promotes tumor cell mitosis by modulating FGF2 in breast cancer (24). In addition, downregulation of GPC-1 attenuated TGF-β1 signaling and Smad2 phosphorylation to suppress pancreatic cancer cell growth (25, 26). GPC-1 also regulates the PTEN/Akt/β-catenin pathway to promote the aggressive proliferation of ESCC cells (27). The above results help us understand that GPC-1 is an unfavorable prognostic factor for cancers. Lu’s study found that high levels of GPC-1 in tumor tissues were associated with poorer differentiation and larger tumor diameters in patients with PDAC (28). Some studies have reported that GPC-1 is highly expressed in multiple human cancer tissues, such as pancreatic cancer (28), breast cancer (29), glioblastoma (30), and ESCC (9), and overexpression of GPC-1 in tumor tissues was found to be associated with shorter OS in patients with PC (10, 28), ESCC (9), breast cancer (29), and glioma (11). Consistent with previous results, the expression of GPC-1 mRNA and protein was significantly increased in pancreatic cancer tissues and associated with shorter RFS of PDAC based on public databases.

In recent years, serum GPC-1+ exosomes have received much research interest in the diagnosis of early-stage pancreatic cancer. Melo’s study (13) reported that GPC-1+ serum exosomes could distinguish PDAC and HCs with a perfect AUC of 1.0 with a sensitivity of 100% and a specificity of 100%. After that, Hu’s (31) study found that the ROC curve of GPC-1 mRNA in serum EVs also showed a perfect AUC of 1.0 in differentiating stage I–IV PDAC patients from HCs and BPD patients. Buscail’s study (32) showed that the diagnostic accuracy reached 0.78 (sensitivity 64% and specificity 90%) when peripheral and portal blood CD63+GPC-1+ exosomes were combined. However, it cannot distinguish between resectable PDAC and intraductal papillary and mucinous neoplasms (IPMNs). Xiao’s study (33) showed that exosomal GPC-1, CD82, and serum CA19-9 could effectively distinguish PC from HCs with an AUC of 0.942 and distinguish PC from CP with an AUC of 0.958. Lucien’s (34) results showed that only GPC-1+ exosomes or combined glycoprotein 2 (GP2) were unable to effectively distinguish between BPD and PC, with AUCs of 0.5404 and 0.5229, respectively. Frampton’s (35) finding showed that crExos GPC-1 could not discriminate between PDAC and benign pancreatic disease (IPMN, CP, and serous cystadenoma). Zhou’s study (36) showed that GPC-1 in total serum could distinguish patients with early-stage PDAC from HCs, BPT patients and CP patients with an AUC of 0.756, which was lower than that of CA19-9 with an AUC of 0.881. Thus, it remains highly controversial whether GPC-1 could be a diagnostic marker for PDAC. In addition, the methods for testing GPC-1+ serum exosomes that the above researchers adopted were different, and all the methods used would be difficult to perform in a standard hospital laboratory, including ultracentrifugation, flow cytometry, and novel nanoparticles. In this study, we attempted to use a simple and available clinical method. ELISA is a simple, efficient, sensitive, and economical practicable approach and is widely used in clinical laboratories. We extracted exosomes from serum using a commercially available exosome isolation kit. Serum exosomal and total serum GPC-1 were found to be detectable by ELISA. Serum exosomal GPC-1 levels were significantly increased compared with GPC-1 levels in total serum and exosome-depleted serum supernatant. These results demonstrated that GPC-1 in serum was mainly derived from exosomes. In addition, we investigated whether repeated freeze–thaw cycles affected GPC-1 stability. The study showed that GPC-1 expression levels in serum exosomes and serum remained stable for up to five repeated f/t cycles. These results indicate that serum exosomes and serum GPC-1 could be potential candidates for the diagnosis and prognosis of PDAC. In addition, GPC-1 was found in higher concentration in the serum exosome fraction compared to total serum and supernatant, which in favor of a strategy that serum exosomal GPC-1 analysis might be more sensitive to analyze GPC-1 than using total serum.

This study showed that GPC-1 can differentiate early-stage PDAC from HCs, and the AUC of the combination GPC-1 and CA19-9 was better than that of GPC-1 or CA19-9 alone, which was consistent with the results of Melo’s study (13). However, GPC-1 levels in CP and BPT were slightly lower than those in PDAC, which did not distinguish PDAC from CP and BPT (AUCs of 0.568 and 0.615), consistent with the results of Lucien’s and Frampton’s studies (34, 35). In previous studies, GPC-1 expression levels significantly decreased compared with the preoperative levels at 2-14 (36), 7 (13), and 28-82 (35) days after surgery, while Xiao’s study (33) showed that the rates of positive exosomal GPC-1 expression were not decreased after surgery. In our study, the expression levels of GPC-1 at 5 days after surgery were elevated compared with those before surgery. Interestingly, serum exosomal and serum GPC-1 levels were also elevated after pancreatic resection in patients with CP and BPT. Our results showed that GPC-1 was highly expressed when the pancreas was under pathophysiological and surgical trauma conditions. We surmised that there are some probable reasons for these findings. First, differences in patient ethnicity and disease stages among different studies contributed to the different results. Second, several studies have indicated a possible protumorigenic role for high GPC-1 expression (11, 26, 27). However, Qiao‘s study (37) showed that moderate GPC-1 overexpression could stimulate glioma blood vessel endothelial cell (EC) growth, but proliferation was inhibited when GPC-1 was either knocked down or overexpressed. Quach‘s study (38) also found a paradoxical effect on cells with a low expression of GPC-1. Low GPC-1 expression in PC-3 cells decreased cell growth and migration *in vitro*, while it increased cell proliferation and migration in DU-145 cells. These results provide some biological plausibility to our clinical observation results. However, the molecular mechanisms of action underlying GPC-1 in pancreatic diseases need to be further explored. Finally, we selected the time points to detect GPC-1 on the 5th day after the operation, and the time was shorter compared to that in the previous literature. Longer follow-up studies are needed to assess whether GPC-1 expression levels decrease to normal levels over time after surgery.

Melo’s report showed that GPC-1+ exosomes in serum were associated with poor OS and disease-specific survival (DFS) (13), and Zhou’s study showed that high levels of serum GPC-1 could also predict poor OS in PDAC patients (36). Our findings agreed with those previous results that serum exosomal and serum GPC-1 were associated with poor RFS. In addition, the initial recurrence time was associated with poor prognosis in PDAC. Yamamoto et al. (39) found that patients with early recurrence had a 9% 5-year survival rate compared with a 42% rate for patients with late recurrence. Groot’s study showed that patients with PDAC who experienced recurrence after surgery within 12 months had a postrecurrence survival (PRS) of 6.1 months compared with 10.8 months for patients with recurrence after 12 months (40). However, there are currently no available factors for predicting early-stage PDAC recurrence after surgery (41). In our study, postoperative serum exosomal and serum GPC-1 levels were independent predictors of early recurrence in patients with PDAC. Hence, serum exosomal and serum GPC-1 analysis might help identify those patients with a high likelihood of early recurrence, which could help physicians select suitable sequences of therapies and develop personalized surveillance strategies.

The limitations of this study were that this was a single-center study with a small sample size. Nevertheless, trends have already become apparent. That is, either serum exosomal or serum GPC-1 was elevated in PDAC and could accurately distinguish early-stage patients from HCs. Whether GPC-1 could differentiate between benign and malignant pancreatic tumors needs to be further validated with a large sample size in multiple centers. It is also worth noting that during exosome isolation step, the commercially available, polymer-based method can introduce large proteins into the precipitated pellet, masking the exosome surface proteins and making a sensitive detection more difficult. This dilemma applies to detection of serum exosomal GPC-1, too. Though we have detected GPC-1 in serum exsomes with more sensitivity compared to whole serum, in the coming validation in large samples, adding an enhanced elution step may make the detection of protein target, especially GPC-1, more specific and prominent.

## Conclusion

In conclusion, the study indicates that ELISA could be an effective method for the detection of serum exosomal GPC-1. Serum exosomal GPC-1 was significantly elevated in patients with pancreatic tumors compared with HCs, especially in PDAC. Serum exosomal GPC-1 could distinguish PDAC from HCs, but it was not able to discriminate between benign and malignant pancreatic diseases. Serum exosomal and serum GPC-1 expression levels were independent predictors of early recurrence and metastasis for early-stage PDAC after surgery.

## Data availability statement

The original contributions presented in the study are included in the article/**Supplementary Material**. Further inquiries can be directed to the corresponding author.

## Ethics statement

This study was reviewed and approved by the Harbin Medical University Cancer Hospital Ethics Committee. The patients/participants provided their written informed consent to participate in this study.

## Authors contributions

JZ, YG and XH conceived and designed the study. JZ, MG and YS collected the samples and worked on the experiment. SL, RL and YuZ performed statistical analysis and wrote the manuscript. YumZ and QY reviewed and edited the manuscript. All authors contributed to the article and approved the submitted version.

## Funding

This work was supported by Haiyan Fund of Harbin Medical University Cancer Hospital (Grant No. JJZD2022-02), CSCO-Hengrui Tumor Research Fund (Y-HR2020ZD-0361), Health Commission of Heilongjiang (2019-060), Health Commission of Zhejiang (2020KY1049) and Project of Taizhou science and Technology Bureau (1901ky46).

## Conflict of interest

The authors declare that the research was conducted in the absence of any commercial or financial relationships that could be construed as a potential conflict of interest.

## Publisher’s note

All claims expressed in this article are solely those of the authors and do not necessarily represent those of their affiliated organizations, or those of the publisher, the editors and the reviewers. Any product that may be evaluated in this article, or claim that may be made by its manufacturer, is not guaranteed or endorsed by the publisher.
