# Supplementary material for: Serum exosomal and serum glypican-1 are associated with early recurrence of pancreatic ductal adenocarcinoma
Source: Front Oncol. 2022 Oct 14;12:992929. doi: 10.3389/fonc.2022.992929 (PMC9614098; doi:10.3389/fonc.2022.992929)
Supplement: Supplementary file 1 [file Table_1.docx]

| Features | Serum exo GPC-1 | | | Serum GPC-1 | | |
| --- | --- | --- | --- | --- | --- | --- |
|  | <1.778 | ≥1.778 | P value | <1.603 | ≥1.603 | P value |
| Sex |  |  | 0.833 |  |  | 0.336 |
| Male | 11(57.9) | 17(54.8) |  | 19(61.3) | 9(47.4) |  |
| Female | 8(42.1) | 14(45.2) |  | 12(38.7) | 10(52.6) |  |
| Age |  |  | 0.944 |  |  | 0.608 |
| ≤60 | 9(47.4) | 15(48.4) |  | 14(45.2) | 10(52.6) |  |
| >60 | 10(52.6) | 16(51.6) |  | 17(54.8) | 9(47.4) |  |
| CA19-9 |  |  | 0.481 |  |  | 0.923 |
| ≥37 | 11(57.9) | 21(67.7) |  | 20(64.5) | 12(63.2) |  |
| <37 | 8(42.1) | 10(32.3) |  | 11(35.5) | 7(36.8) |  |
| Location: |  |  | 0.740 |  |  | 0.344 |
| Head | 12(63.2) | 21(67.7) |  | 22(71.0) | 11(57.9) |  |
| Body or Tail | 7(36.8) | 10(32.3) |  | 9(29.0) | 8(42.1) |  |
| Tumor size |  |  | 0.702 |  |  | 0.287 |
| >4cm | 4(21.1) | 8(25.8) |  | 9(29.0) | 3(15.8) |  |
| ≤4cm | 15(78.9) | 23(74.2) |  | 22(71.0) | 16(84.2) |  |
| Differentiation |  |  | 0.382 |  |  | 0.771 |
| High and Moderate | 11(57.9) | 14(45.2) |  | 15(48.4) | 10(52.6) |  |
| Poor | 8(42.1) | 17(54.8) |  | 16(51.6) | 9(47.4) |  |
| Nerve invasion |  |  | 0.849 |  |  | 0.849 |
| Yes | 13(68.4) | 22(71.0) |  | 13(68.4) | 22(71.0) |  |
| No | 6(31.6) | 9(29.0) |  | 6(31.6) | 9(29.0) |  |
| Lymph nodes |  |  | 0.702 |  |  | 0.326 |
| Yes | 4(21.1) | 8(25.8) |  | 6(19.4) | 6(31.6) |  |
| No | 15(78.9) | 23(74.2) |  | 25(80.6) | 13(68.4) |  |
| TNM stage |  |  | 0.667 |  |  | 0.879 |
| I | 11(57.9) | 16(51.6) |  | 17(54.8) | 10(52.6) |  |
| II | 8(42.1) | 15(48.4) |  | 14(45.2) | 9(47.4) |  |

Supplementary Table 1 Correlation between GPC-1 and clinicopathological features in PDAC
