# Supplementary material for: Serum exosomal and serum glypican-1 are associated with early recurrence of pancreatic ductal adenocarcinoma
Source: Front Oncol. 2022 Oct 14;12:992929. doi: 10.3389/fonc.2022.992929 (PMC9614098; doi:10.3389/fonc.2022.992929)
Supplement: Supplementary file 2 [file Table_2.docx]

Supplementary Table 2 Univariate analysis of GPC-1 for RFS in patients with PDAC

| Characteristics | HR (95% CI) | P value |
| --- | --- | --- |
| Sex (male vs. female) | 1.239(0.529-2.900) | 0.621 |
| Age (>60 vs.≤60) | 0.665(0.284- 1.557) | 0.347 |
| Location (head vs. body, tail) | 0.699(0.299-1.638) | 0.410 |
| Stage (II vs. I) | 1.461(0.632-3.374) | 0.375 |
| Lymph node metastasis (yes vs. no) | 2.104(0.852-5.195) | 0.107 |
| Differentiation (poor vs. moderate and well) | 1.324(0.573-3.058) | 0.511 |
| Nerve invasion (yes vs. no) | 0.935(0.381-2.297) | 0.883 |
| CA19-9(≥37 vs. <37U/ml) | 1.078(0.452-2.570) | 0.866 |
| Tumor size (>4 vs. ≤4cm) | 1.032(0.404-2.638) | 0.947 |
| Serum GPC-1 (≥1.603 vs. <1.603ng/ml) | 2.738(1.164-6.438) | 0.021 |
| Serum exo GPC-1(≥1.778 vs. <1.778 ng/ml) | 3.782(1.274-11.228) | 0.017 |
