# Supplementary figures and images for: Serum exosomal and serum glypican-1 are associated with early recurrence of pancreatic ductal adenocarcinoma
Source: Front Oncol. 2022 Oct 14;12:992929. doi: 10.3389/fonc.2022.992929 (PMC9614098; doi:10.3389/fonc.2022.992929)

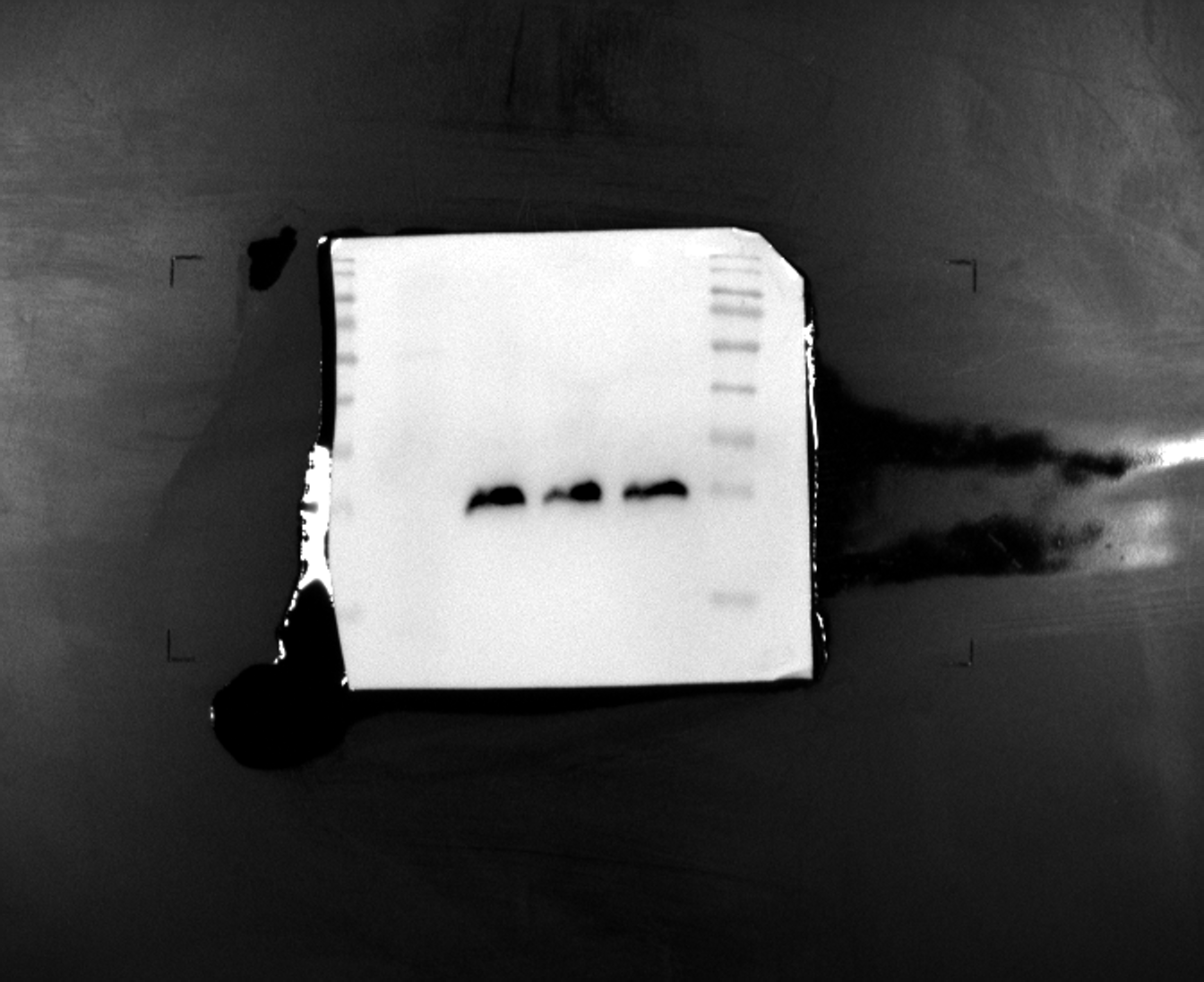

Supplement: Supplementary file 3 [file Image_1.tif]

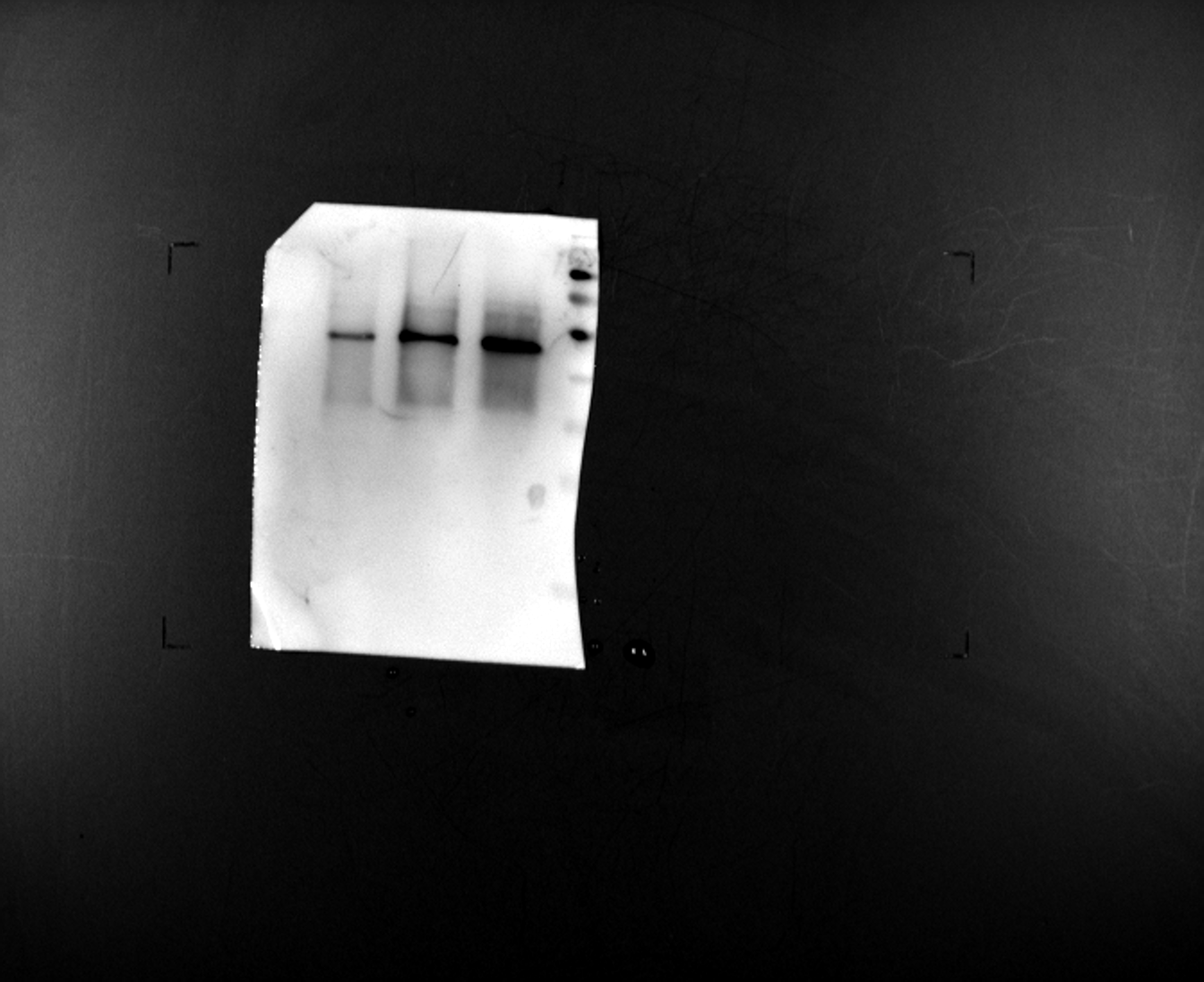

Supplement: Supplementary file 4 [file Image_2.tif]

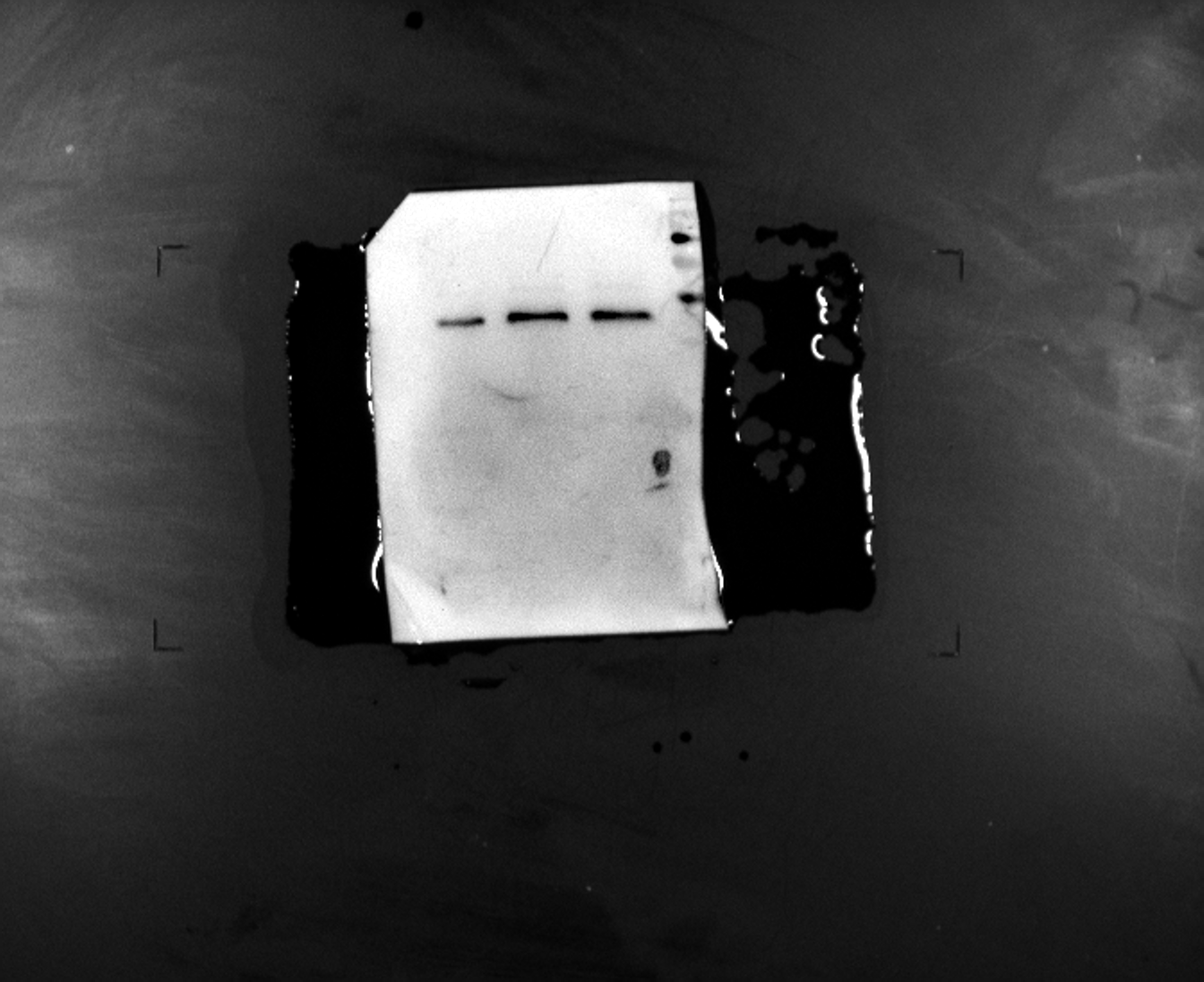

Supplement: Supplementary file 5 [file Image_3.tif]

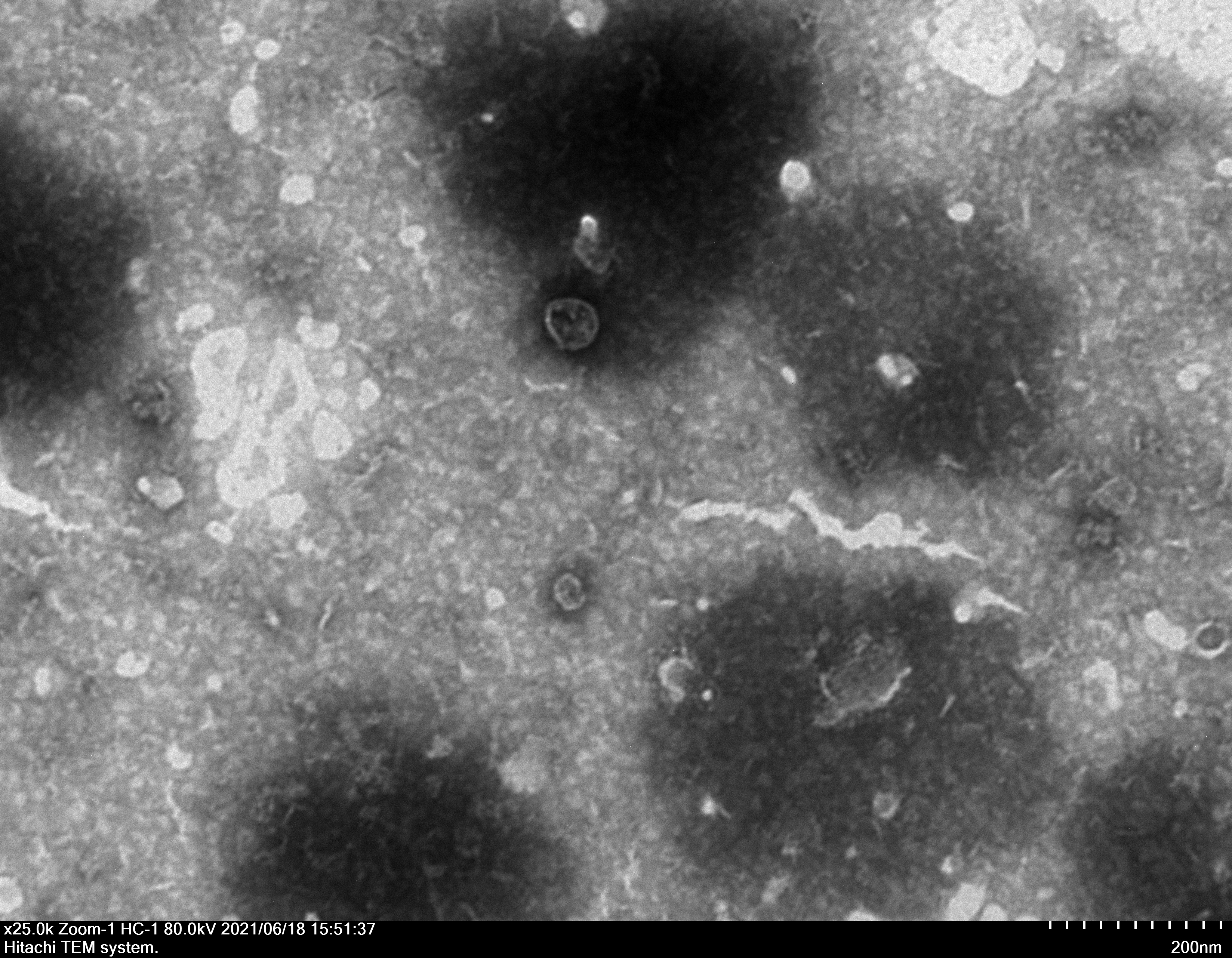

Supplement: Supplementary file 6 [file Image_4.jpeg]
